# Optimising door-to-needle-time in children with febrile neutropenia in the paediatric emergency unit—a quality improvement study

**DOI:** 10.3332/ecancer.2026.2063

**Published:** 2026-01-20

**Authors:** Jyothi Muni Reddy, Lavanya Rajkumar, Shivangi Bora, G M Vanitha, Deepa Eapen, Ranjini Srinivasan

**Affiliations:** 1 Department of Paediatric Haematology Oncology, St. John’s Medical College Hospital, Bangalore 560034, Karnataka, India; 2 Department of Paediatrics, St. John’s Medical College Hospital, Bangalore 560034, Karnataka, India; 3 Department of Emergency Medicine, St. John’s Medical College Hospital, Bangalore 560034, Karnataka, India; a https://orcid.org/0000-0001-8092-7882

**Keywords:** antibiotics, children, clinical care pathway, febrile neutropenia, paediatric emergency unit, quality improvement, root cause analysis

## Abstract

**Introduction and Background:**

Febrile Neutropenia is one of the leading causes of treatment-related mortality in children with malignancies. Door-to-needle-time (DTNT), defined as the time between arrival at hospital and antibiotic administration, of <60 minutes is considered standard of care in managing this oncological emergency.

**Objectives:**

In this quality improvement (QI) study, we aim to determine the proportion of children with febrile neutropenia (FN) receiving timely antibiotics in the Paediatric Emergency unit and improve this by 50% over 6 months using the Plan-Do-Study-Act framework. Secondary objectives included identifying factors associated with delays in achieving optimal DTNT and the impact of these delays on clinical outcomes.

**Methods:**

Baseline data of children less than 18 years of age on cancer chemotherapy presenting with neutropenic fever between January 2022 and June 2023 were collected ambispectively. A fishbone root cause analysis was performed. Interventions were planned and prioritised using a prioritisation matrix. QI measures were initiated subsequently in the form of the development and implementation of a validated clinical care pathway that described the triaging, clinical assessment, laboratory investigations, antibiotic dosing and patient disposition. Additionally, mannequin-assisted, followed by bedside training on chemoport handling, was performed. Post-intervention data, including the 6-months sustenance phase, were collected, and outcomes were analysed.

**Results:**

In the baseline, intervention and sustenance periods, 129, 80 and 47 episodes of FN, respectively, were documented. Median DTNT reduced by 50% from a baseline of 80 minutes interquartile range (IQR 65–105) to 40 minutes (IQR 30–40) post-intervention (*p* < 0.001). The proportion of children with optimal DTNT increased from 22% to 92.5% (*p* < 0.001). DTNT > 60 minutes was significantly associated with intensive care admissions. Root-Cause-Analysis revealed difficult peripheral venous access, lack of awareness and implementation challenges as the most important causes for delays in antibiotic administration.

**Conclusions:**

The median DTNT was significantly delayed in patients with FN. Inadequate knowledge and skills training, delays in vascular access and lack of implementation of standard protocols were identified as the key factors responsible for delays in antibiotic administration. QI strategies aimed at improving awareness and skill with rigorous training programs as well as clinical pathway implementation, significantly improved the time to antibiotic administration.

## Introduction

Globally, an estimated 400,000 children and adolescents develop cancer each year. Low- and middle-income countries (LMICs), including India, contribute more than 90% of this burden [1]. According to a recent report of the National Cancer Registry Program in India, childhood cancers account for 5.5% of all cancers [2]. Mortality in children with cancer is higher in LMICs compared to high-income countries (HICs), with treatment-related mortality (TRM) being 45% in LMICs compared to less than 5% in HICs [3]. Sepsis is the leading cause of TRM, constituting more than 70% of these deaths [3]. Sepsis-related mortality in children with cancer is more than twice that seen in children without cancer [4]. A recent international Delphi consensus study has identified sepsis management as a top research priority to improve paediatric onco-critical care outcomes [5].

Febrile neutropenia (FN), the most common oncological emergency occurring in up to 30% of neutropenic episodes in children undergoing cancer chemotherapy, predisposes them to sepsis and thereby considerable morbidity and mortality [6]. It is considered a medical emergency because patients who often appear clinically stable may deteriorate rapidly within a short period if antibiotics are not initiated promptly. One of the cornerstones in reducing morbidity and mortality in patients with FN is the timely administration of the first dose of antibiotic. Prolonged door-to-needle-time (DTNT), defined as the time from arrival at the hospital to antibiotic administration, can adversely impact outcomes in these patients [7, 8]. The International Paediatric Fever and Neutropenia Guideline Panel recommends administering the first dose of antibiotic as soon as possible, ideally within 60 minutes of arrival at the emergency department, particularly for children with high-risk FN [9].

DTNT as a quality indicator was initially employed in the management of community-acquired pneumonia, meningitis and septic shock in adults [8]. This was later extrapolated in the setting of emergency management of FN patients despite a lack of data on its impact on outcomes [7, 8, 10]. A systematic review by Koenig *et al* [10] found that delays in antibiotic delivery seemed to be associated with impaired ‘safety’, defined as death, Intensive care unit (ICU) admission and severe sepsis. This, however, had a strong influence of triage bias and other confounding factors.

Although several quality improvement (QI) projects aimed at reducing DTNT in children with FN have been published in the literature, data from a developing country like India are lacking [11]. This study aimed to improve the median baseline DTNT and thereby the proportion of children with potential FN receiving timely antibiotics in the paediatric emergency unit (PER) using a QI framework and to analyse its impact on outcomes. The primary objective was to determine the proportion of children with FN receiving timely antibiotics in PER and to improve this proportion by 50% over a period of 6 months using the Plan-Do-Study-Act (PDSA) methodology. The secondary objectives were to identify various factors associated with delay in antibiotic administration and to study the impact of delays in DTNT on immediate outcomes of children with FN.

## Methods

A QI initiative with a quasi-experimental, interventional study design was implemented in the PER unit of a tertiary teaching institute in January 2022 after obtaining approval from the Institutional Ethics Committee. The SQUIRE 2.0 guidelines have been utilised to write the methodology.

### Setting and context

The PER is a 13-bedded unit, including an isolation cubicle that caters to all medical and surgical emergencies in children under 18. Patients from paediatrics and various paediatric subspecialties with acute and chronic illnesses requiring emergency care, including oncological emergencies, are managed in the PER and are supported by the paediatric and neonatal ICUs. It forms a division of the department of paediatrics in a 1,350-bed teaching hospital with 90 paediatric beds, including a 22-bed intermediate treatment unit for managing sicker patients. The PER operates 24 hours a day on all days of the week and is managed by junior and senior residents as well as faculty in the department of paediatrics. Nurses trained in emergency care, including paediatric emergencies, rotate in three shifts and work in liaison with emergency physicians. The emergency handles an influx of about 30–40 patients a day, which may increase in numbers based on seasonal disease trends.

The Department of Paediatric Hematology and Oncology consists of an independent 30-bed patient unit that includes a daycare center and is run by specialised faculty, fellowship trainees and junior residents posted in the department. The department manages patients with benign hematological conditions as well as various lymphoreticular and solid organ malignancies, supported by departments of paediatrics, paediatric surgery and paediatric and neonatal ICUs. The department also offers stem cell transplantation for malignancies and non-malignant conditions such as bone marrow failure, primary immune deficiencies and histiocytic disorders.

Around 100 children are newly diagnosed every year with lymphoreticular or solid organ malignancy. As part of standard protocol, all patients undergoing chemotherapy have a central venous access device (CVAD), usually a chemoport, inserted during hospital stay, which is accessed by the oncology nurse for chemotherapy drug infusion. These children are sent home with the device *in situ* after caregiver training regarding chemoport care is completed. As part of the discharge process, caregivers are also educated about FN and the need to immediately seek emergency care in case of fever.

Approximately 3–5 such children on cancer chemotherapy with FN are seen every month in the PER unit. The patients are triaged in accordance with the South African Triage System, which has been adopted by our center [12]. A child with suspected FN, if stable, is usually seen within 15–30 minutes in a busy PER and assessed by the resident posted in that shift. Following triage and initial assessment, blood samples are obtained preferably by peripheral vascular access. This is usually performed by a PER nurse. Accessing the chemoport in the PER was avoided for fear of central line-associated bloodstream infection (CLABSI) due to handling by untrained staff. The time taken to obtain samples and insert a peripheral venous access device varies considerably and depends on several factors including patient characteristics such as age, nutritional status, previous intravenous punctures, body mass index and difficult intravenous access (DIVA) score, nurse related factors including training and expertise of the person drawing the samples as well as other determinants such as staffing in PER and the number of existing patients. This, in turn, determines the DTNT for antibiotic administration.

### Intervention

A multidisciplinary QI team comprising an emergency physician, paediatricians, a paediatric oncologist, a paediatric oncology trainee. An emergency nurse and an oncology nurse was formed to initiate this QI project. Children between 1 month and 18 years of age on cancer chemotherapy presenting with potential FN, to the PER, were included in the study. Those children who had received parenteral antibiotics before arrival at the PER were excluded.

### Study definitions

- Potential FN: A case of potential FN was defined as a child receiving myelosuppressive drugs for haematological/solid tumours presenting with a single recording of fever (temp > 38.30°C or 101°F) or temperature greater than 38°C (100.4°F) sustained over 1 hour [13].

Baseline data on patient demography, underlying oncological diagnosis, treatment details, FN episodes in the past, focus of infection and details regarding investigations were obtained by reviewing medical records ambispectively, i.e., retrospectively between January 2022 and December 2022, and prospectively from January 2023 to June 2023. The time of arrival at the PER and the time of administration of the first dose of antibiotic for every patient were noted by reviewing the PER register and the patient chart, respectively. The time of antibiotic administration was recorded by the PER nurse, who was not part of the study during the period of data collection. This was cross-checked for accuracy by noting the time the blood culture sample was sent, which was verified from the Health Management Information System as corroborating evidence. The DTNT was then computed from the time difference. In addition, patient-related outcomes such as need for

Paediatric Intensive Care Unit (PICU) admission, duration of hospital stay, duration of PICU stay, mortality and cost of hospitalisation incurred were also documented.

The following outcome measures were chosen to study the impact of interventions conducted:

- DTNT: The time from arrival at the PER to administration of the first dose of antibiotic was considered as DTNT [7, 8]. The target DTNT for antibiotic administration in children with potential FN was determined to be within 60 minutes.
- The proportion of children who received antibiotics within 60 minutes was computed and a specific goal to improve this proportion from baseline by more than 50% over a period of 6 months was set.

From the baseline data collected, it was observed that there was a considerable delay in antibiotic administration, with DTNT exceeding 60 minutes in a large proportion of children. Additionally, the impact of delays in antibiotic administration on clinical outcomes was determined by case record analysis.

### Root cause analysis

**Figure 1.**
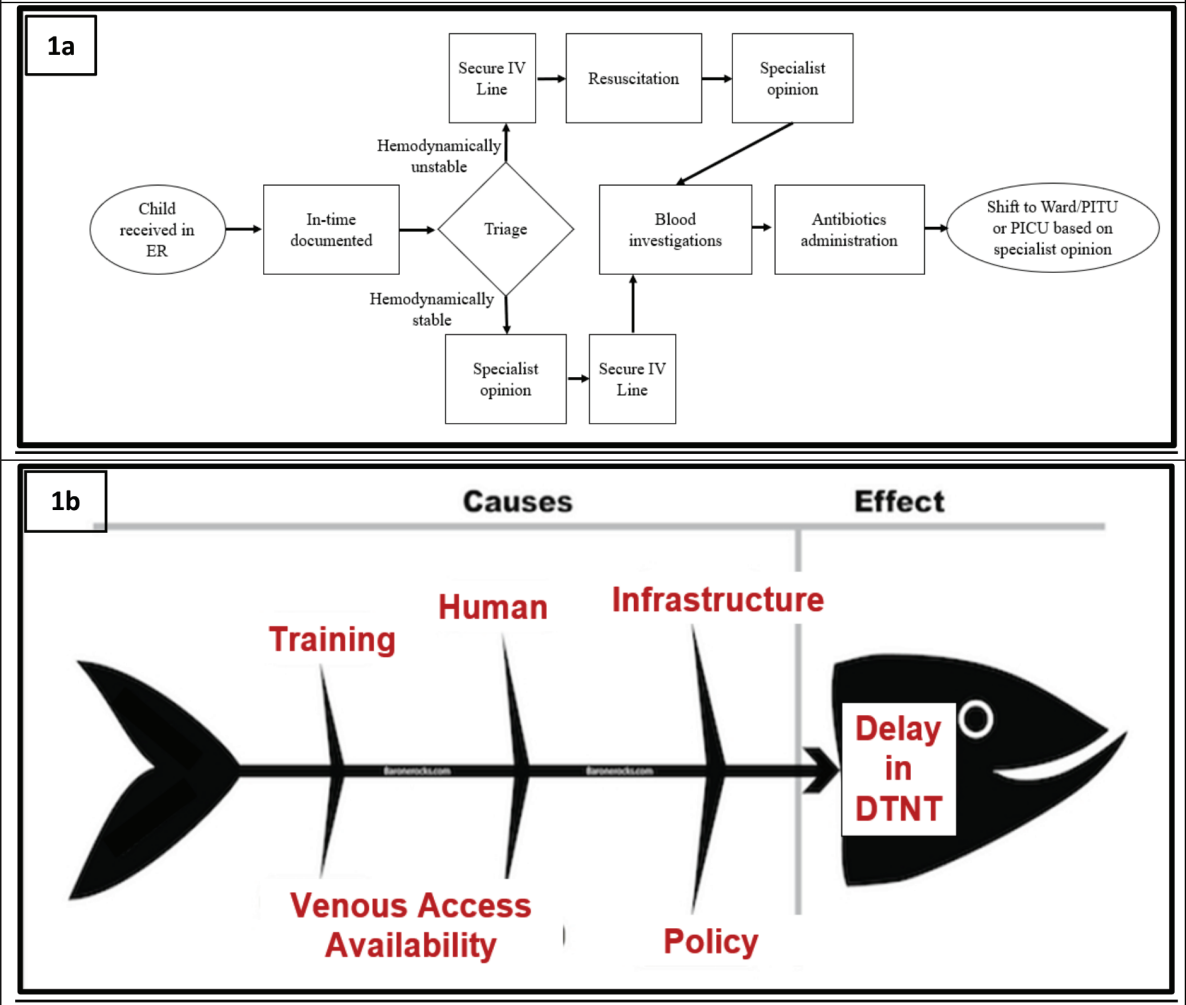
Root cause analysis. (a): process flowchart, (b): fishbone analysis.

A process flowchart, as described in Figure 1a, was developed. All healthcare providers (HCPs), i.e., nurses, paediatric residents and faculty involved in treating children with FN in the PER were requested to identify steps in the flowchart contributing to delays in antibiotic administration as well as factors contributing to such delays. These data were procured by conducting interviews, small group discussions and online surveys. The data were consolidated using a fishbone diagram (Figure 1b), and interventions planned (Table 1) were prioritised using the prioritisation matrix (Table 2). All the causes of delayed DTNT were listed. Each of these was scored on a scale of five concerning four parameters – importance, affordability, measurability and modifiability. Scoring was done based on the relative value of one process/outcome over the other. A cut-off score ≥ 12 was given maximum priority based on which interventions were planned and implemented during PDSA 1, as indicated by the red boxes in Table 2.

### QI Strategies

PDSA cycles were initiated after identifying factors contributing to delayed antibiotic administration. Those processes scoring highest on the prioritisation matrix were targeted first as described below. These included designing and implementation of a clinical care pathway (CCP), identification of a designated isolation area in the PER to receive patients with FN, increasing awareness among doctors and nurses by periodic training sessions comprising didactic lectures and hands-on skills training to enable the HCPs to access the chemoport for timely antibiotic administration. The immediate impact of interventions in PDSA 1 in the form of outcome measures listed previously was documented and analysed over the subsequent 3-month period.

### PDSA 1

This period of intervention was executed over a 3-month period between July and September 2023.

#### STEP 1: Establishment of an independent isolation cubicle in the PER

**Figure 2.**
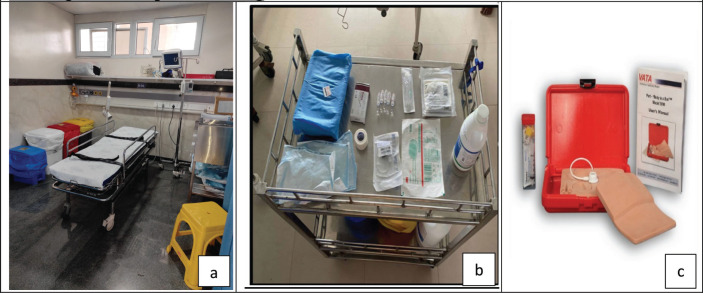
Interventions implemented. (a): isolation cubicle, (b): chemoport access trolley, (c): VATA Inc.’s ‘Body in a box’ 5010 model used for training on chemoport handling.

A dedicated standard air pressure isolation room or cubicle for receiving children with FN was established. The creation of this space avoided delays due to waiting time for beds in PER and offered the benefit of contact isolation from other children with infections received in the regular orange/red zone area. The isolation room was equipped with a trolley bed, multi-parameter monitor, nursing trolley, crash cart for resuscitation and drug infusion pumps (Figure 2a and b).

#### STEP 2: Development of CCP

The CCP was a step-by-step guide in the form of a time-determined flow chart that included triage, focused history and examination, steps for chemoport handling, details about antibiotic dosages and infusions, investigations to be ordered and final patient disposition. The CCP was a ready reckoner that was simplified and incorporated standard guidelines from literature. Recommendations on empirical antibiotics were based on antibiograms and hospital/paediatric oncology department policy. This pathway was developed by the QI team and reviewed and validated by experts in paediatric oncology, emergency medicine and nursing departments. Posters describing the pathway were displayed in the isolation room. Additionally, hard copies of the CCP with an in-built simplified system for documentation of clinical findings using ‘✓’ for positive findings and ‘✗’ for negative findings to reduce time invested in documentation were made available for use (Refer Annexure 1).

#### STEP 3: Execution of CCP and skills training

To ensure effective use of the CCP, training of nurses and doctors in the following aspects was conducted:

1. Recognition of FN as an emergency
2. Golden hour management of FN using CCP (Annexure 1)
3. Hand hygiene (HH) & aseptic non-touch technique (ANTT)
4. Chemoport insertion and maintenance bundle (Annexure 2)
5. Execution of multiple medication orders using 3-way stopcocks and extension tubings.

These training sessions were conducted by the QI team for all the nurses and doctors working in PER. Reading material on infection control, HH, CCP flowchart and FN management guidelines developed by the Indian Academy of Paediatrics was provided 1 week before the training session [14]. Four training sessions were conducted (two in each PDSA cycle). A pre-test was held before each session. These sessions lasted 3 hours each and included lectures on the aspects mentioned above, with interactive participation and demonstration of chemoport needle insertion and maintenance using a mannequin. The mannequin used was VATA Inc.’s ‘Body in a box’ 5,010 model (Figure 2c), which is a simulation mannequin of the paediatric chest with a chemoport *in situ*, allowing for needle insertion, aspiration of blood-coloured dye, flushing and clamping. The bundle checklist for chemoport insertion and maintenance was also discussed (Annexure 2). This checklist was displayed in the isolation cubicle for guidance during central line (CL) handling. A post-test was provided after the session to evaluate the impact of the training session (Annexure 3).

#### STEP 4: Chemoport handling in the emergency

Chemoport handling included chemoport needle insertion bundle (also referred to as chemoport access bundle) and maintenance bundle. A nurse or doctor trained in chemoport needle insertion from the paediatric oncology department was deputed every shift for inserting the chemoport needle/accessing the chemoport in PER. However, after chemoport needle insertion, HCPs in PER were expected to handle the CL by adhering to the maintenance bundle. This arrangement was made to avoid the risk of CLABSI, as nurses in the PER did not have experience with chemoport needle insertions.

#### STEP 5: Redefining nursing responsibilities during shift change

To circumvent delays arising from nursing duty shifts, HCPs caring for a child with FN were expected to complete antibiotic administration before handing over responsibilities to another HCP.

### PDSA 2

This took place between October and December 2023 and included the following interventions.

#### STEP 6: Repeated training to enable chemoport access in the PER by the physician or nurse

Dependence on an oncology nurse for chemoport needle insertion was seen as a barrier to optimal execution of the CCP. Hence, doctors and nurses in the PER underwent periodic simulation-based training on chemoport needle insertion. During this period, they were posted for 1 hour daily, on rotation, in the oncology day-care ward to obtain bedside training in chemoport access. This was achieved in three phases – observation of chemoport access, access chemoport with assistance and access chemoport independently but under the supervision of an oncology day-care nurse. This was documented by the HCPs in their academic logbook (Annexure 5). HCPs were considered competent and allowed to access chemoports in PER if they completed two observations, two insertions with assistance and two insertions independently with >80% adherence to the checklist.

#### STEP 7: Setting up the equipment trolley for chemoport access

Preparation of equipment for chemoport needle insertion during the golden hour was considered a challenge in adhering to the CCP and timely antibiotic administration in PDSA 1. Hence, a fully equipped chemoport access trolley was arranged, and it was checked once during every shift by the senior nurse. This measure considerably reduced the time taken for chemoport access.

#### STEP 8: Availability of restricted antibiotics in the PER

Restricted antibiotics were usually stocked in the main hospital pharmacies, which were not easily accessible to the PER. These antibiotics were not allowed to be stocked in the PER to avoid antibiotic misuse as per the policy of the Hospital Infection Control Committee. However, they were made available in the PER pharmacy, which was situated a few metres away from the PER. This significantly decreased the delays associated with drug procurement.

#### STEP 9: Overcoming attrition-related problems

Constant attrition and rotation of nurses resulted in incomplete skills training in chemoport access. Hence, multiple training sessions at regular intervals for newly employed HCPs were organised. A list of HCPs posted in the PER for every month was noted. Untrained HCPs were identified, and training either before or within 2 weeks of posting in the PER was ensured. The impact of the training session was assessed using pre-test and post-test scores as described earlier. However, overcoming HCP attrition was a constant barrier faced by the QI team that was often difficult to overcome, considering the number of repeated training sessions required.

The assessment of processes developed in PDSA 1 & 2 was done using process indicators (Annexures 4 and 6) that were identified and defined at the start of the intervention period and measured after interventions were carried out.

**Sustenance phase:** This period extended from January 2024 to June 2024. The gains achieved through the previous interventions were consolidated in the sustenance phase.

**Sample size determination & statistical analysis:** Sample size was calculated to estimate the proportion of children receiving antibiotics within 1 hour of reaching the healthcare facility. Considering 50% adherence to antibiotic timing (i.e., 50% of the children do not receive the first dose of antibiotics on time based on the study by Gonzalez *et al* [15], to observe an improvement (irrespective of baseline) of 50%

with 20% relative precision and 95% confidence interval, the number of subject participants needed was 97. The formula used was *n* = *z*^2^*pq*/*d*^2^, where *n* is the estimated minimum sample size, *z* is the level of significance set at 95% confidence, *p* is the expected prevalence of the characteristic being studied, *q* is (1−*p*) and *d* is the margin of error. The *p*-value was computed using the chi-square test. *p*-value <0.05 was considered significant.

## Results

**Figure 3.**
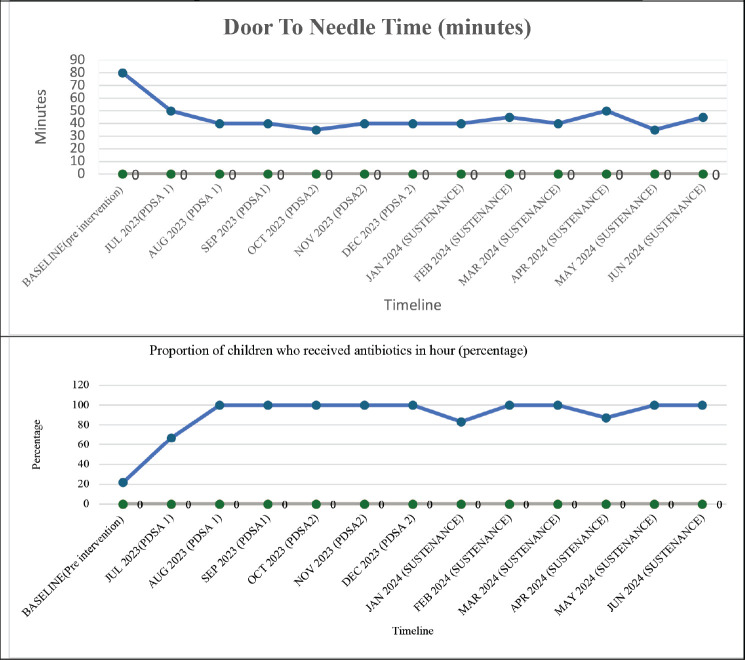
Run charts of outcome measures. (a): Median Door-to-needle-time in minutes, (b) proportion of children who received antibiotics in 1 hour.

In the baseline study period, 129 episodes of FN in 92 children were analysed. 44 & 36 episodes of FN were documented during each PDSA cycle, respectively, and 47 episodes during the sustenance phase. Table 3 compares the clinical characteristics of the baseline, intervention and sustenance cohorts. The groups were homogeneous in all aspects. The majority of children had haemato-lymphoid malignancies. All patients had a CVAD or chemoport. Table 4 compares the process indicators and outcome measures between baseline and post-intervention groups. The median DTNT reduced by 50% from a baseline of 80 minutes interquartile range (IQR 65–105) to 40 minutes (IQR 35–50) post-intervention (*p* < 0.001). The proportion of children who received antibiotics within 60 minutes of hospital entry increased from a baseline of 22%–86% after the 1st PDSA cycle and 100% after the 2nd PDSA cycle (*p* < 0.001). Chemoport was accessed to administer antibiotics in all children in the post-intervention period, as compared to only 19% at baseline (*p* < 0.001). No CLABSIs were reported in the post-intervention groups attributable to CVAD handling in PER. Figures 3a and b represent the run charts showing a steady and progressive decline in the median DTNT and a significant increase in the proportion of children receiving timely antibiotics after initiating PDSA cycles and during the sustenance phase. No patients required admission to the intensive care in the post-intervention group, against a baseline of 10% (*p* = 0.002). The length of hospital stay and cost incurred differed significantly between the two groups (Table 4). Table 1 summarises the interventions carried out in the PDSA cycles. In this cohort of 256 episodes of FN, DTNT greater than 60 minutes was associated with higher mortality, cost of hospitalisation, PICU admissions and inotrope requirement on univariate analysis. On adjusting for age, gender and hemodynamic status, DTNT was significantly associated with need for PICU admissions (*p* value = 0.016).

## Discussion

Neutropenic fever in children can result in significant morbidity and mortality if not managed appropriately. Improving time to antibiotic administration (TTA) is considered one of the measures in providing quality care to patients in oncology settings in developed countries [16–21]. However, robust data from LMICs are limited and only started emerging recently [22–24]. Although standard guidelines recommend empirical antibiotic administration within 60 minutes of triage [9, 14], studies from LMICs report varied results for DTNT due to factors at various levels that may influence this outcome [22, 23].

A prospective analysis of children (*n* = 211) with FN in Chandigarh, India, reported that TTA within 60 minutes was achieved only in 66% of children [22]. Through root cause analysis, physician unawareness, waiting for blood counts, DIVA and delays in antibiotic procurement and preparation were identified as causes for delays in this cohort. On the other hand, a 3-month audit conducted in a tertiary hospital in Kolkata showed that the expected target of DTNT within 60 minutes was met in more than 80% of children. However, the causes for delays were not analysed [23]. A systematic review on adult and paediatric studies that assessed the effectiveness of various interventions aimed at reducing DTNT in oncology centers in HICs revealed that the percentage reduction in TTA varied between 22% and 73% [11]. Factors associated with delays included lack of awareness, absence of triage protocol for managing FN, delays in physician assessment, challenges in securing venous access, long turnaround time for dispensing drugs, unavailability of laboratory results, nurses' workflow issues and lack of established protocols [11]. The results of the current study, which included 256 FN episodes in 191 children, demonstrated that before interventions, only 22% of patients with FN received timely antibiotics, with key factors associated with delays including physician unawareness, problems with intravenous access, lack of an established CCP to streamline patient management, excessive documentation and delay in dispensing antibiotics. Table 5 compares this study with other relevant paediatric QI projects available in the literature, describing the patient cohorts, mean DTNTs, percentage improvement in DTNTs and various interventions implemented to achieve set targets.

The present study demonstrated a significant reduction in the median DTNT time from 80 to 40 minutes after various interventions at different time points were initiated and implemented. One of the key interventions in the current study was the development of a validated CCP. This pathway empowered PER to initiate antibiotics early, minimising delays typically caused by consulting existing protocols, calculating drug doses, referencing infusion guidelines, waiting for oncological consultations and awaiting blood test results. As a result, it enhanced awareness and timely management of this medical emergency.

A QI project that evaluated the effectiveness of a rapid TTA pathway for patients with FN in an ambulatory infusion center found that the TTA reduced from a mean of 79.6 (±40.4) minutes in the pre-pathway group (*n* = 16) to 41.2 (±23.9) minutes in the post-pathway group (*n* = 9) (*p* = 0.0068) [28]. Other outcome improvements noted included reduction in mean time from lab order to lab results (63.25–27 minutes) and lab result to antibiotic administration time (49–7.5 minutes) [28]. Likewise, another multiphase QI project developed in Saudi Arabia to improve TTA reported a significant reduction in DTNT from 3 hours 48 minutes to 1 hour 15 minutes following the implementation of CCP [19].

The decreasing time to therapy (DoTT) project was developed to reduce TTA by utilising the World Health Organisation multimodal improvement strategy model [24]. This project incorporated several targeted interventions, including the development of a healthcare delivery bundle and antibiotic selection pathways, called ‘Build It’. A structured training initiative called ‘Teach it’ was implemented to educate emergency HCPs. To ensure adherence, the ‘Check it’ component monitored the application of the DoTT bundle. Lastly, the ‘Sell it’ strategy involved distributing educational materials, such as posters and pamphlets, as reminder tools for HCPs [24].

The CCP developed in this study included triaging patients with FN, defining criteria to identify those with circulatory insufficiency requiring immediate care, having a rapid documentation sheet for relevant history and examination and guiding the emergency physician regarding appropriate investigations and antibiotics. Moreover, it ensured that all patients presenting with FN were managed uniformly according to a standardised protocol, thereby eliminating observer variations.

Another important intervention in the current study was to enhance the skill set of physicians and nurses and enable them to access the chemoport device for antibiotic administration. This intensive training, which was provided by senior oncology nurses and oncology physicians, eliminated delays associated with multiple attempts made to secure peripheral venous access, as was done in the pre-intervention cohort. Similarly, the QI model in Saudi Arabia also found that implementation of a nurse-led clinical pathway and having ‘Nurse Champions’ helped in improving skills regarding CVAD, which played a pivotal role in the outcomes observed [19].

Comparably, in a protocol-based study that aimed at reducing TTA by training emergency department nurses on the use of subcutaneous venous access devices (SCADs) for antibiotic delivery, the mean DTNT significantly decreased from 96.9 ± 57.8 minutes to 64.3 ± 28.4 minutes (*p* < 0.0001) following implementation of the protocol [30].

PICU admissions in our cohort dropped to zero in the post-intervention and sustenance period. Increasing awareness among HCPs, effective implementation of the CCP and accessing the chemo port to administer antibiotics were probably the measures undertaken that effectively reduced the requirement for ICU. Salstrom *et al* [8] studied the impact of QI interventions on TTA and clinical outcomes. They observed that the need for ICU reduced by 20% (*p* = 0.003) when a TTA of less than 60 minutes was achieved. A retrospective cohort analysis found that 60-minute TTA intervals were associated with composite adverse events outcomes that included PICU admission, fluid resuscitation and mortality [7].

This study demonstrated a significant reduction in the median length of hospital stay (50%), and the total cost incurred from hospitalisation, on comparing the pre- and post-intervention cohorts (*p* < 0.001). Salstrom *et al* [8] reported a decrease in the median length of stay in the hospital in the maintenance phase when compared to the study period data, although the results were statistically insignificant.

Certain studies, however, had conflicting results and found that TTA below 60 minutes had no impact on clinical outcomes. A prospective multicentre study (*n* = 266 FN episodes) investigated the association between TTA and safety-relevant events (SRE). Interestingly, poorer outcomes were associated with shorter TTA, while a trend toward increased risk of SRE with longer TTA was observed only in patients with severe disease (*n* = 36; rate ratio 2.02, 95% CI: 0.34–12.06). This counterintuitive finding was attributed to triage bias, where more severely ill patients received antibiotics more quickly upon arrival. Additionally, since all patients reached the hospital within three hours of fever onset, outcomes may have been favourable regardless of TTA. These results may not be generalisable to our setting, where patients often travel longer distances and face greater delays in accessing care. Furthermore, the study focused solely on SREs and did not evaluate other relevant outcomes such as length of hospital stay or cost of care [32].

Another study from the USA evaluated whether a TTA metric <60 minutes was associated with favourable clinical outcomes in paediatric FN. The study observed that the median TTA in those requiring PICU admission was 28 minutes (IQR: 20, 37), and TTA ≥ 60 minutes was not associated with any complication [33]. However, this study was again conducted in medical centers where CLs were routinely accessed to rapidly administer antibiotics to those who appeared ill and where educated caregivers could access emergency services quickly to seek medical attention. Therefore, the findings of these studies may not be generalisable to centers in LMICs [33].

A study from Australia showed that TTA < 60 minutes from hospital triage showed no impact on risk of adverse outcome or prolonged hospital stay [34]. It was postulated that antibiotic administration within the ‘golden hour’ would benefit only those with bacteremia, i.e., 10%–15% of all FN patients, suggesting that a nuanced approach to FN management would be to identify this group with bacteremia by the timely execution of sepsis screening by trained medical professionals. However, the study failed to establish a causal association between delayed TTA and adverse outcome, even when stratified for bacteremia-risk prediction or for established bacteremia [34]. Also, in this study, the overall median TTA was 53 minutes, which was within the recommended time of 60 minutes. Therefore, this may have resulted in the reduction in the incidence of adverse outcomes studied overall. Moreover, the median time from fever onset to TTA was 135 minutes, which also impacted outcomes. In a low-middle-income setting such as ours, the median time from fever onset to TTA varies considerably. A study done in a well-networked setting may underestimate the challenges faced in LMICs. Factors such as malnutrition, crowding, poor sanitation and poor access to health care increase infection risk and complicate comparisons with high-income cohorts. Therefore, risk stratification models for bacteraemia may not be comparable because baseline vulnerabilities differ. This may limit the applicability of the results of the above study.

To the best of our knowledge, this is the first paediatric QI project on FN carried out in India. Most studies in the subcontinent have been observational and outcomes after interventions have not been analysed. Although complete blinding was not possible, training and assessments were carried out by an independent group who were not part of the QI team, thereby minimising observer bias. The interventions carried out in the form of establishing a CCP and training HCPs in the PER can be done in other similar centers as well. The regular debriefing sessions conducted as part of training and periodic feedback collected from caregivers and health care personnel helped in improvisation. Repeated emphasis on HH, regular infection control audits and regular appraisals ensured that accessing the chemoport in the PER did not increase the risk of acquiring CLABSIs.

However, there were some limitations to this study. Although statistically, the sample size seemed adequate, a larger post-intervention cohort may be required to give better results when adjusted for covariates. Moreover, the number of seriously ill patients was very small, and therefore, it may have been challenging to correlate TTA with the occurrence of adverse events. Constant attrition and the recruitment of new staff were significant barriers to the training sessions. Few interventions, like accessing the chemoport for antibiotic administration, may not be feasible in all PERs as it requires considerable training and supervision.

## Conclusion

In this QI study, the authors were able to improve the average DTNT from 80 to 40 minutes and the proportion of children receiving antibiotics within the golden hour from 22% to 100%. This was achieved through the PDSA methodology, wherein interventions that were high priority based on the prioritisation matrix were executed in PDSA 1. The impact and problems associated with these interventions were identified and corrected in PDSA 2. Development of a CCP, accessing chemoports in PER, and repeated training of HCPs were the key interventions that helped achieve these outcomes. Delay in antibiotic administration >60 minutes was associated with significantly higher PICU admissions. This QI model is implementable and sustainable, as demonstrated in this study.

## Novelty statement

What is already known on this topic: Door-to-needle time is a well-established quality indicator for the management of febrile neutropenia in children on cancer chemotherapy.

What this study adds: Quality improvement strategies such as the development of clinical care pathway, chemoport access in the emergency unit and repeated training of health care professionals can improve door-to-needle time and sustain the benefits achieved even in resource-limited settings.

How this study might affect research, practice or policy: This quality improvement (QI) model can serve as the framework for implementing multicentric QI projects in low- & middle-income countries.

## Abbreviations

CCP: Clinical Care Pathway, CLABSI: Central line-associated bloodstream infection, CVAD: Central venous access device, DIVA: Difficult intravenous access, DoTT: Decreasing Time to Therapy, DTNT: Door-to-needle time, FN: Febrile neutropenia, HCPs: Healthcare providers, HH: Hand Hygiene, HICs: High-income countries, HMIS: Health Management Information System, LMICs: Low- and middle-income countries, PDSA: Plan-Do-Study-Act, PER: Paediatric Emergency Unit, PICU: Paediatric Intensive Care Unit, QI: Quality Improvement, SCAD: Subcutaneous venous access devices, SRE: Safety-relevant events, TRM: Treatment-related mortality, TTA: Time to antibiotic administration.

## Conflicts of interest

The authors declare no conflicts of interest.

## Funding

None.

## Author contributions

JMR and RS: Conceived, designed and conducted the study. DE and VGM implemented the clinical care pathway and trained nurses and doctors in chemoport handling. LR and SB collected and analysed the data. JMR, RS and SB drafted the paper. RS revised the manuscript for important intellectual content. The final manuscript was approved by all authors.

## Data availability statement

Data supporting the findings of this study are available on request from the authors.

## Institutional Ethics Committee (IEC) study reference number

207/2022.

## Figures and Tables

**Table 1.** Factors causing delayed DTNT and interventions planned.

Causes of delayed DTNT	Interventions planned and implemented
Area 1: Infrastructure	
Lack of an isolation room for management of FN	A dedicated isolation space for receiving children with FN was identified and set up with the equipment required to manage such patients.
Area 2: Training	
Lack of awareness regarding FN management	Periodic training sessions to improve awareness about FN, including the importance of golden hour management, were conducted.
Streamlining care of children with febrile neutropenia and avoiding variations in management of this condition among emergency physicians.	Development of a clinical care pathway that was to be implemented by all doctors and nurses following training. Periodic training sessions to enhance awareness and knowledge regarding the management of FN in the PER
Unnecessary delays caused by waiting for confirmatory laboratory reports or an oncologist consult before initiation of antibiotics.	
Lack of training in the safe handling of Central Venous Access Device (CVAD)	Mannequin and bedside skills training and certification on chemoport access and maintenance bundle
Area 3: Policy	
Delays in patient care during nursing shift changes	Nursing responsibilities during shift change were redefined and priorities outlined. In PDSA 2, nurses were expected to prepare the chemoport access equipment trolley prior to every shift.
Excess time consumed by clinical documentation in medical records by HCPs	A rapid documentation sheet was made available for this
Avoidance of CVAD handling in emergency unit (ER) due to the risk of CLABSI, due to handling by untrained staff	To initiate chemoport access and maintenance in PER without compromising asepsis and safety. (In PDSA 1, an oncology nurse was deputed for chemoport access. In PDSA 2, the HCPs in PER were trained to access the chemoport)
Flaws in simultaneous execution of multiple medication orders. Ex: In those with shock, antibiotics were administered after intravenous (IV) fluid bolus instead of simultaneous administration	Training and utilisation of 2-way or 3-way stopcocks and extension tubings were carried out
Restricted antibiotics were not stocked in PER	Restricted antibiotics were made available in PER by written requests to hospital pharmacies.
Area 4: Intravenous access	
Difficult peripheral IV cannulation	Doctors and nurses underwent multiple trainings followed by assessments in chemoport handling to enable rapid antibiotics administration via chemoport, thereby reducing delays caused by failed attempts at peripheral vascular access.
Parental refusal of peripheral IV cannulation due to the presence of chemoport	
Area 5: Personnel	
Attrition of nurses and constant rotation of ER residents	Regular and repeated training was carried out to overcome the attrition barrier.


CLABSI: Central line-associated bloodstream infection, CVAD: Central venous access device, DTNT: Door-to-needle time, FN: Febrile neutropenia,

HCP: Healthcare provider, PER: Paediatric Emergency Unit

**Table 2.** Prioritisation matrix.

Interventions planned	Importance	Affordable	Measurable	Controllable	Total score
To establish a dedicated isolation space for receiving children with FN	5	1	5	3	14
Training to improve awareness of the golden hour management of FN	5	5	3	5	18
Development of a clinical care pathway for management of FN in the PER	5	5	3	5	18
Skills Training on chemoport access and maintenance bundle	4	4	3	3	14
Redefine nursing responsibilities during shift change	3	5	4	2	14
Rapid documentation sheet	2	5	5	5	17
Training and utilisation of two-way or three-way stopcocks and extension tubings	5	5	1	3	14
Stock restricted antibiotics in PER	1	1	5	1	8
To initiate chemoport access and maintenance in PER without compromising asepsis and safety to avoid peripheral vein cannulation	5	3	5	3	16
Regular and repeated training to counter attrition	3	2	3	2	10

Note: Red colour indicates high priority interventions, while yellow indicates low priority interventions

FN: Febrile neutropenia, PER: Paediatric Emergency Unit

**Table 3.** Comparisons of variables in pre-intervention and post-intervention groups.

Baseline variable	Pre-intervention	Post-intervention	Sustenance data	*p* value
No. of FN episodes	129	80	47	--
No. of children with FN	92	64	35	--
Median age in months (IQR)	48 (30, 96)	36 (30, 84)	48 (33, 69)	0.30
Males, *n* (%)	72 (56)	43 (54)	28 (60)	0.76
Type of diagnoses, *n* (%) Hematolymphoid cancers Solid tumours	114 (88.3) 15 (11.7)	75 (93.75) 5 (6.25)	43 (91.5) 4 (8.5)	0.16
Median time since last chemotherapy in days (IQR)	7 (4, 10)	5 (2, 10)	5 (2, 10)	0.07
Median duration of fever prior to arrival at PER in hours (IQR)	4 (2, 8)	5 (2, 8)	4 (2, 4)	0.34
No. of FN episodes with hemodynamic instability, *n* (%)	17 (13.1)	4 (5)	5 (10.6)	0.58
% of children with CVAD	100	100	100	
Median absolute neutrophil count at admission (IQR)	1,100 (550, 1,800)	1,200 (742.5, 1,785)	1,100 (440, 1,500)	0.55
No. of children requiring restricted antibiotics, *n* (%)	17 (13.1)	4 (5)	5 (10.6)	0.58
No. of children with positive blood cultures, *n* (%)	14 (10.85)	2 (2.5)	5 (10.6)	0.07

CVAD: Central venous access device, FN: Febrile neutropenia, PER: Paediatric Emergency Unit

**Table 4.** Process indicators and outcome measures.

	Baseline (*n* = 129)	PDSA 1 (*n* = 44)	PDSA 2 (*n* = 36)	Sustenance (*n* = 47)	*p* value
**a: Process indicators**					
Adherence to the CCP*, *n* (%)	NA	44 (100)	36 (100)	44 (93.6)	--
Adherence to HH*, (%)	NA	31/40 (77.5)	28/40 (70)	34/47 (72.34)	--
No. of children whose CL (Chemoport) was accessed in ER, (%)	24 (18.6)	44 (100)	36 (100)	47 (100)	<0.001
Adherence to chemoport maintenance bundle^*^ (%)	NA	12/15 (80)	10/15 (66.66)	22/30 (73.33)	--
Adherence to chemoport access bundle^*^ (%)	NA	NA	11/15 (73.33)	24/30 (80)	--
*Denominator refers to the total number of audits performed in that period					
**b: Outcome measures**					
Median DTNT in minutes (IQR)*	80 (65,105)	40 (35,50)	40 (30,40)	40 (32.5,55)	<0.001
No. of children who received antibiotics in 1 hour, (%)	28 (21.7)	38 (86.36)	36 (100)	45 (95.75)	<0.001
No. of patients requiring PICU care, (%)	13 (10.08)	0 (0)	0 (0)	0 (0)	0.002
Median duration of antibiotics in days (IQR)*	10 (10,14)	5.5 (5,7)	5 (5,6)	5 (4.5,7)	0.02
Median length of hospital stay in days (IQR)*	10 (10,14)	7 (5,10)	6 (5,7)	5 (5,7)	0.022
Median cost incurred in rupees (IQR)*	47,100 (33,135, 66,942.50)	34,520 (21,367.50, 65,250.00)	29,178 (22,724.25, 35,529.75)	28,800 (22,700, 39,267)	<0.001


CCP: Clinical Care Pathway; DTNT: Door-to-needle time; HH: Hand Hygiene; PICU: Paediatric Intensive Care Unit

**Table 5.** Summary of paediatric QI projects aimed at improving DTNT.

Authors	Year published	Country	FN episodes (patients)	Improvement in average DTNT pre- and post-intervention (minutes)	Improvement in percentage (%) adherence to golden hour antibiotics pre- and post-intervention	QI strategy
Mendieta *et al* [24]	2023	Peru	137 (129)	146 to 69	--	DoTT multimodal strategy – ‘Build it, Teach it, Check it, Sell it and Live it’
Gonzalez *et al* [15]	2021	Mexico	204 (105)	67.5 to 35	50 to 88	Antibiotic availability at point of care, FN guideline development, education, auditing and monitoring, mentoring, and dissemination
Kram *et al* [17]	2020	USA	401 (401)	58 to 28	51 to 96	Interventions to overcome delays in patient triage, antibiotic ordering, antibiotic choice, and bedside indwelling Port-a-Cath accessing procedure
Lukes *et al* [18]	2019	USA	101 (101)	128 to 53	0 to 83	Improving provider and nursing workflow, improving nurse autonomy
Maddi Pole *et al* [19]	2019	Saudi Arabia	104	228 to 75	--	Development of clinical care pathway & nursing training related to efficient CVAD handling
Emerson *et al* [20]	2019	USA	80	116 to 55	--	Secure text-based messaging platform, creating a new antibiotic pathway, and educating staff and family.
Sudairy *et al* [21]	2019	Saudi Arabia	358	255 to 49	--	Improving the triaging process, creating an electronic ‘chemotherapy alert caution” and order sets for physicians, and using the hot-line by nurses to call the pharmacy to expedite the process of preparation of antibiotics.
Monroe *et al* [25]	2018	USA	--	--	40 to 80	Streamlining triage process, staff notification at patient arrival, rapid port access, nurse champions on every shift, and departmental awareness of progress
Yoshida *et al* [26]	2018	USA	718 (327)	83 to 65	47 to 69	Rapid absolute neutrophil count test and clinical standardised work pathway
Spencer *et al* [27]	2017	USA	1032 (1032)	118.5 to 57 (site 1) 163 to 97.5 (site 2), and 188 to 111.5 (site 3)	--	1) Triage application of topical anesthetic 2) Rapid room placement & triage 3) Resuscitation room placement of ill appearing children 4) Close proximity to CL equipment 5) Antibiotic administration before laboratory analyses 6) Consensus clinical practice guideline establishment 7) Family pre-admission education and 8) Staff project updates
Vanderway *et al* [28]	2017	USA	25 (25)	79.6 to 41.2	--	Implementation of rapid time-to-antibiotics pathway
Benner *et al* [29]	2016	USA	253 (111)	207 to 88.5	1 to 31	Implementation of clinical practice guideline (CPG) and prearrival antibiotic ordering and preparation
Cohen *et al* [30]	2016	USA	253 (253)	96.9 to 64.3	35 to 51.4	Formulation and implementation of protocol for FN management that addressed delays in CL access, antibiotic order, and pharmacy delivery
Salstrom *et al* [8]	2015	USA	116 (116)	134 to 43	19 to 74	Provider awareness, rapid appointments and laboratory results, designated rooms, antibiotic prescription & stocking
Volpe *et al* [31]	2012	USA	365 (365)	99 to 49	50 to 88.5	A multidisciplinary team approach and standardisation of the process of care


CVAD: Central venous access device, DoTT: Decreasing Time to Therapy, DTNT: Door-to-needle time, FN: Febrile neutropenia, QI: Quality Improvement

## Annexure 1. Clinical care pathway for febrile neutropenia.

### Any child (1 month–18 years) presenting to ER with potential febrile neutropenia

Focussed History in Emergency

1. Underlying diagnosis: ______________________________________________
2. Duration of fever: ________________________________ hours
3. Transplant recipient? Yes/No
4. If yes: When was transplant done?
5. Associated symptoms: Trace focus - Tick one or more
  **Table:**
  System involved	Tick relevant box
  • Respiratory	
  • Oropharyngeal/mucositis	
  • Gastrointestinal tract	
  • Genito-urinary tract	
  • Skin and soft tissue	
  • CL site infection	
  • Others	
6. Date of last chemotherapy:
7. Chemotherapy regimen used currently:
8. Febrile neutropenia in the past: If yes, how many episodes?
9. Symptomatic household contact:
10. Underlying co morbidities: Yes/No
11. Culture reports and antibiogram of previous admission if available

Relevant Physical Examination

1. Hemodynamic Stability: stable/unstable
2. ABC: Physiological derangement- Tick ✓ appropriate option
  - Respiratory distress: Yes/No
  - Hypoxia: Yes/No
  - Shock: Compensated/Hypotensive
  - Altered sensorium: Yes/No
3. Temperature:
4. Last Urine output
5. Focused examination for evidence of infection: Tick the appropriate box
  **Table:**
  All CVC sites		Respiratory System	
  Mucositis		Abdomen	
  GUT examination		Skin/soft tissue sites	
  Perianal region for ulcers		Surgical site tenderness	

Final Diagnosis:

Disposition: Ward/ITU/PICU (Tick one✓) –Refer to Appendix 3

Laboratory Investigations to be sent from Emergency: Tick ✓ against whatever has been sent

1. Complete blood count
2. Blood culture from peripheral line
3. Blood culture from CL (all lumens)
4. CRP/procalcitonin
5. Serum electrolytes, GRBS
6. Urine routine/culture
7. Chest Radiograph
8. Skin swabs for culture
9. Swabs from inflamed mucous sites for culture
10. Others if any:

## Appendix 1. Steps to access chemo port device (to be done in isolation room).

**Table:**

Sl. No	Observation categories (Tick when done)
Part 1	2 HCP/staff assistance present HCP 1: Junior nurse/Ward PG HCP 2: Senior nurse/SR/ER-PG
Part 2	HCP 1: Chemoport access trolley to be moved to patient side. Proceed iff no port site infection
Part 3	HCP 2/Senior nurse/PG: Pre procedure standard precautions
1.	Don cap & mask, perform hand wash
2.	Use standard precautions (Sterile gown & sterile gloves)
Part 4	HCP 1: Prepare tray (While HCP 2 at step 3a)
1.	Place sterile dressing tray
2.	Place required articles in tray with help of HCP: Gauze, gloves, syringes, huber needle, chlorhexidine, hepsaline, tegaderm, micropore, prilocaine)
Part 5	HCP 2: Insertion procedure
1.	Skin preparation done with 2% Chlorhexidine x 3 times using 3 guazes for 15 seconds each time followed by 15 seconds drying
2.	Place glove cover near the field of action
3.	Load 10cc syringe (1) with hepsaline, flush huber line and clamp
4.	Position huber needle and insert
5.	Unclamp
6.	Withdraw for backflow Collect samples for blood culture and other investigation if backflow present using syringe (2). Do not delay antibiotic administration for sample collection. Administer antibiotics Flush with hepsaline
7.	Clamp
8.	Dressing with tegaderm
Part 6	HCP 2: Post procedure precautions
1.	Waste disposal
2.	Hand hygiene (HW) performed

Note: Chemoport access kit & trolley should be ready for use at the start of the day

Note: Chemoport access kit includes:
Tray containing sterile cup, guaze holder, guaze, Other components: Sterile gown, sterile gloves, cap, mask, gauze, chlorhexidine solution, hepsaline, tegaderm, huber needle, 10cc syringes (2)

## Appendix 2. Antibiotics for emergency management of febrile neutropenia.

1. Ceftazidime: 50mg/kg/dose divided q8 hourly (max dose 6g/day) 170mg/ml concentration in NS/dextrose containing fluids over 3-5 minutes or infusion (1-40mg/ml) over 15-30 minutes.
*Caution: Do not mix with aminoglycoside*
2. Amikacin: 15- 20mg/kg/day once daily as infusion over 30 minutes (Max concentration 10mg/ml) in NS/Dextrose containing fluids/RL (max dose 1500 mg/day) Caution: Do not administer as an IV push 3. Meropenem:40mg/kg/dose divided q8 hourly (max dose 6g/day) IV push over 3-5 minutes: max concentration 50mg/ml in NS/dextrose containing fluids Intermittent infusion over 15 minutes: max concentration 50mg/ml in NS/dextrose containing fluids 4. Vancomycin: 15mg/kg/dose q6 hourly (max 4g/day) Intermittent infusion over 60 minutes at a maximum concentration of < 5mg/ml. Can be diluted in NS/RL/Dextrose containing fluids.
*Caution: Do not administer as IV push*
Indications for empiric vancomycin

- Hemodynamic instability
- Skin/catheter site infection(cellulitis/abscess)
- Suspect staphylococcal pneumonia on chest radiograph
- Severe mucositis if fluoroquinolone prophylaxis has been given

## Annexure 2. CL maintenance bundle.

**Table:**

Sl No	Observation categories	Status			Remark	Action plan
		Yes	No	NA		
Part 1	Medication loading					
1.	Perform hand rub					
2.	Open tray					
3.	Use Box gloves					
4.	Load medications using ANTT					
5.	Cover tray					
6.	Place tray near patient					
Part 2	Pre-line handling Preparation					
7.	Unclamp the CL					
8.	Open dressing over hub exposing hub					
Part 3	Medication administration					
9.	Perform hand wash					
10.	Use sterile gloves before procedure					
11.	Place glove cover over the chest/near CL					
12.	Rub the hub with chlorhexidine for 15 seconds and dry for 15 seconds					
13.	Flush the line with 10 cc NS followed by 5 cc hepsaline before medication. If blood withdrawn prior to line flush, use 20cc NS followed by 5 cc hepsaline					
14.	Follow safe injection and infusion practices					
15.	Follow ANTT throughout medication administration procedure					
16.	Flush line again post medication					
Part 4	Post medication administration					
17.	Is CL dressing changed					
18.	Discard wastes generated during procedure					
19.	Perform hand hygiene after procedure					

### Chemoport needle insertion bundle

**Table:**

Sl No	Observation categories	Status			Remark	Action plan
		Yes	No	NA		
Part 1	2 HCP/staff assistance present					
Part 2	Prepare trolley					
1.	Clean trolley with alcohol					
2.	Place objects on trolley					
Part 3	Pre procedure precautions					
3.	Perform hand wash					
4.	Use maximal sterile barrier precautions (mask, gown, sterile drape and sterile gloves)					
Part 4	Prepare tray					
5.	Place sterile dressing tray					
6.	Place required articles in tray with help of HCP					
Part 5	Insertion procedure					
7.	Skin preparation done with 2% Chlorhexidine × three times using three guazes for 15 seconds each time followed by 15 seconds drying					
8.	Place glove cover near the field of action					
9.	Flush line and clamp					
10.	Position huber needle and insert					
11.	Unclamp					
12.	Flush after withdrawal for backflow					
13.	Clamp					
14.	Dressing with tegaderm					
Part 6	Post procedure precautions					
15.	Waste disposal					
16.	Hand hygiene (HW) performed					

Scrub the hub protocol: Scrub the sides (threads) and end of the hub thoroughly with friction, making sure to remove any residue (e.g., blood). Using the same antiseptic pad, apply antiseptic with friction to the catheter, moving from the hub at least several centimeters towards the body. Hold the limb while allowing the antiseptic to dry. Use a separate antiseptic pad for each hub/ catheter limb. Leave hubs ‘open’ (i.e., uncapped and disconnected) for the shortest time possible.

## Annexure 3. Febrile neutropenia questions- pre/post test.

1. Which of following regarding ANTT is false

a. ANTT protects key parts and key sites from the health care worker and procedure environment

b. Aseptic field management protects key parts and sites from immediate procedure environment

c. Aseptic field management is the same for surgical and standard ANTT

d. Hand hygiene is not required for ANTT

Ans: c and d

2. Identify the wrong statement regarding Gloves usage

a. Gloves must be worn at all times during patient care

b. Sterile gloves must be worn when coming in contact with blood and body fluids

c. Examination gloves can be worn during CL access handling

d. Glove usage always prevent transmission of infections

Ans: All statements a,b,c,d

3. Which of the statements regarding scrub the hub technique is incorrect

a. Scrub the sides (threads) and end of the hub thoroughly with friction, making sure to remove any residue (e.g., blood).

b. Using the same antiseptic pad, apply antiseptic with friction to the catheter, moving from the hub at least several centimeters towards the body.

c. Hold the hub while allowing the antiseptic to dry

d. Scrub the hub atleast for 30 seconds and dry for 30 seconds

Ans: c and d

4. Which of the following regarding CL insertion bundle is true

a. Skin preparation should be done using only alcohol for atleast 30 seconds each time for a total three times using different swabs

b. Always flush the line after accessing chemoport

c. Use 2cc or 5cc syringes to flush the line with NS or hepsaline.

d. Line flush required only if blood draw involved during procedure

Ans: b

5. Which of the following is the main route of cross-transmission of potentially harmful germs between patients in a health-care facility?

A. Health-care workers’ hands when not clean

B. Air circulating in the hospital

C. Patients’ exposure to colonised surfaces (i.e., beds, chairs, tables and floors)

D. Sharing non-invasive objects (i.e., stethoscopes, pressure cuffs and so on) between patients

Ans: A

6. What is the most frequent source of germs responsible for health care-associated infections?

A. The hospital’s water system

B. The hospital air

C. Germs already present on or within the patient

D. The hospital environment (surfaces)

Ans: c

7. Which of the following hand hygiene actions prevents transmission of germs to the patient?

A. Before touching a patient

B. Immediately after a risk of body fluid exposure

C. After exposure to the immediate surroundings of a patient

D. Immediately before a clean/aseptic procedure

Ans: a and d

8. Which of the following hand hygiene actions prevents transmission of germs to the health-care worker?

A. After touching a patient

B. Immediately after a risk of body fluid exposure

C. Immediately before a clean/aseptic procedure

D. After exposure to the immediate surroundings of a patient

Ans: a,b and d

9. Which of the following statements on alcohol-based handrub and handwashing with soap and water are true?

a. Handrubbing is more rapid for hand cleansing than handwashing

b. Handrubbing causes skin dryness more than handwashing

c. Handrubbing is more effective against germs than handwashing

d. Handwash and handrub are recommended to be performed in sequence

Ans: a

10. What is the minimal time needed for alcohol-based handrub to kill most germs on your hands? (tick one answer only)

A. 20 seconds

B. 3 seconds

C. 1 minute

D. 10 seconds

Ans: a

11. Hand washing (and not hand rub) is required in the following situations?

a. Before palpation of the abdomen

b. Before giving an injection

c. After emptying a bedpan

d. After removing body fluid soiled examination gloves

e. After making a patient's bed

f. After visible exposure to blood

Ans: c, f, d

12. Which of the following should be avoided, as associated with increased likelihood of colonisation of hands with harmful germs?

A. Wearing jewellery

B. Damaged skin

C. Artificial fingernails

D. Regular use of a hand cream

Ans: a,b,c

13. A child with febrile neutropenia and no hemodynamic compromise should be seen in the emergency within….

a. 10 minutes

b. 15 minutes

c. 20 minutes

d. Immediately

Ans: a

14. Antibiotic of choice for FN patient in shock is

a. Ceftazidime and amikacin

b. Piperacillin tazobactam

c. Piperacillin Tazobactam and vancomycin

d. Meropenem, amikacin and Vancomycin

Ans: d

15. Indications for empiric vancomycin in patients with FN are all except:

a. Hemodynamic instability

b. Skin/catheter site infection

c. Unavailability of ceftazidime

d. Complicated pneumonia with effusion on chest radiograph

Ans: c

## Annexure 4. WHO hand hygiene audit tool.

### General Recommendations

#### (refer to the Hand Hygiene Technical Reference Manual)

In the context of open and direct observations, the observer introduces him/herself to the health-care worker and to the patient when appropriate, explains his/her task and proposes immediate informal feed back.

The health-care worker, belonging to one of the main four following professional categories (see below), is observed during the delivery of health-care activities to patients.

Detected and observed data should be recorded with a pencil in order to be immediately corrected if needed.

The top of the form (header) is completed before starting data collection (excepted end time and session duration).

The session should last no more than 20 minutes (±10 minutes according to the observed activity); the end time and the session duration are to be completed at the end of the observation session.

The observer may observe up to three health-care workers simultaneously, if the density of hand hygiene opportunities permits.

Each column of the grid to record hand hygiene practices is intended to be dedicated to a specific professional category. Therefore numerous health-care workers may be sequentially included during one session in the column dedicated to their category. Alternatively each column may be dedicated to a single health-care worker only of whom the professional category should be indicated.

As soon as you detect an indication for hand hygiene, count an opportunity in the appropriate column and cross the square corresponding to the indication(s) you detected. Then complete all the indications that apply and the related hand hygiene actions observed or missed.

Each opportunity refers to one line in each column; each line is independent from one column to another.

Cross items in squares (several may apply for one opportunity) or circles (only a single item may apply at one moment).

When several indications fall in one opportunity, each one must be recorded by crossing the squares.

Performed or missed actions must always be registered within the context of an opportunity.

Glove use may be recorded only when the hand hygiene action is missed while the health-care worker is wearing gloves.

### Short description of items

**Table:**

Facility:	to complete according to the local nomenclature		
Service:	to complete according to the local nomenclature		
Ward:	to complete according to the local nomenclature		
Department:	to complete according to the following standardised nomenclature:		
	medical, including dermatology, neurology, haematology, oncology and so on.		surgery, including neurosurgery, urology, EENT, ophthalmology and so on.
	mixed (medical & surgical), including gynaecology		obstetrics, including related surgery
	paediatrics, including related surgery		intensive care & resuscitation
	emergency unit		long term care & rehabilitation
	ambulatory care, including related surgery		other (to specify)
Period No:	1) pre- / 2) post-intervention; and then according to the institutional counter.		
Date:	day (dd) / month (mm) / year (yy)		
Start/end time:	hour (hh) / minute (mm).		
Session duration:	difference between start and end time, resulting in minutes of observation.		
Session No:	attributed at the moment of data entry for analysis.		
Observer:	observer’s initials (the observer is responsible for the data collection and for checking their accuracy before submitting the form for analysis.		
Page No:	to write only when more than one form is used for one session.		
Prof.cat:	according to the following classification:		
	1. nurse / midwife	1.1 nurse, 1.2 midwife, 1.3 student.	
	2. auxiliary		
	3. medical doctor	3.1 in internal medicine, 3.2 surgeon, 3.3 anaesthetist / resuscitator / emergency physician, 3.4 paediatrician, 3.5 gynaecologist, 3.6 consultant, 3.7 medical student.	
	4. other health-care worker	4.1 therapist (physiotherapist, occupational therapist, audiologist, speech therapist), 4.2 technician (radiologist, cardiology technician, operating room technician, laboratory technician and so on), 4.3 other (dietician, dentist, social worker and any other health-related professional involved in patient care), 4.4 student.	
Number:	number of observed health-care workers belonging to the same professional category (same code) as they enter the field of observation and you detect opportunities.		
Opp(ortunity):	defined by one indication at least		
Indication:	reason(s) that motivate(s) hand hygiene action; all indications that apply at one moment must be recorded		
	bef.pat: before touching a patient		aft.b.f: after body fluid exposure risk
	bef.asept: before clean/aseptic procedure		aft.pat: after touching a patient
			aft.p.surr: after touching patient surroundings
HH action:	response to the hand hygiene indication(s); it can be either a positive action by performing handrub or handwash, or a negative action by missing handrub or handwash		
	HR: hand hygiene action by handrubbing with an alcohol-based formula HW: hand hygiene action by handwashing with soap and water		Missed: no hand hygiene action performed

### Observation Form – Basic Compliance Calculation

**Table:**

	Facility:								Period:				Setting:					
	**Prof.cat.**				**Prof.cat.**				**Prof.cat. **				**Prof.cat.**			**Total per session**		
**Session No**	**Opp (*n*)**	**HW (*n*)**	**HR (*n*)**	**Opp (*n*)**		**HW (*n*)**	**HR (*n*)**	**Opp (*n*)**		**HW (*n*)**	**HR (*n*)**	**Opp (*n*)**		**HW (*n*)**	**HR (*n*)**	**Opp (*n*)**	**HW (*n*)**	**HR (*n*)**
**1**																		
**2**																		
**3**																		
**4**																		
**5**																		
**6**																		
**7**																		
**8**																		
**9**																		
**10**																		
**11**																		
**12**																		
**13**																		
**14**																		
**15**																		
**16**																		
**17**																		
**18**																		
**19**																		
**20**																		
**Total**																		
**Calculation**	** Act (*n*) =** **Opp (*n*) =**			** Act (*n*) =** **Opp (*n*)** =					** Act (*n*) =** **Opp (*n*) =**			**Act (*n*)** = **Opp (*n*) =**				**Act (*n*)** = **Opp (*n*) =**		
**Compliance**																		
					

**Table:**

Compliance (%) =	Actions	×100
	Opportunities	

### Instructions for use

1. Define the setting outlining the scope for analysis and report related data according to the chosen setting.

2. Check data in the observation form. Hand hygiene actions not related to an indication should not be taken into account and vice versa.

3. Report the session number and the related observation data in the same line. This attribution of session number validates the fact that data has been taken into count for compliance calculation.

4. Results per professional category and per session (vertical):

4.1 Sum up recorded opportunities (opp) in the case report form per professional category: report the sum in the corresponding cell in the calculation form.

4.2 Sum up the positive hand hygiene actions related to the total of opportunities above, making difference between handwash (HW) and handrub (HR): report the sum in the corresponding cell in the calculation form.

4.3 Proceed in the same way for each session (data record form).

4.4 Add up all sums per each professional category and put the calculation to calculate the compliance rate (given in percent)

The addition of results of each line permits to get the global compliance at the end of the last right column.

### Observation Form – Optional Calculation Form (Indication-related compliance with hand hygiene)

**Table:**

	Facility:						Period:			Setting:					
	**Before touching a patient**			**Before clean/ aseptic procedure**			**After body fluid exposure risk**			**After touching a patient**			**After touching patient surroundings**		
**Session No**	**Indic (*n*)**	**HW (*n*)**	**HR (*n*)**	**Indic (*n*)**	**HW (*n*)**	**HR (*n*)**	**Indic (*n*)**	**HW (*n*)**	**HR (*n*)**	**Indic (*n*)**	**HW (*n*)**	**HR (*n*)**	**Indic (*n*)**	**HW (*n*)**	**HR (n)**
**1**															
**2**															
**3**															
**4**															
**5**															
**6**															
**7**															
**8**															
**9**															
**10**															
**11**															
**12**															
**13**															
**14**															
**15**															
**16**															
**17**															
**18**															
**19**															
**20**															
**Total**															
**Calculation**	**Act (*n*) =** **Indic1 (*n*) =**			**Act (*n*) =** **Indic2 (*n*) =**			**Act (*n*) =** **Indic3 (*n*) =**			**Act (*n*) =** **Indic4 (*n*) =**			**Act (*n*) =** **Indic5 (*n*) =**		
**Ratio act / indic^*^**															

### Instructions for use

Define the setting outlining the scope for analysis and report related data according to the chosen setting.

Check data in the observation form. Hand hygiene actions not related to an indication should not be taken into account and vice versa.

If several indications occur within the same opportunity, each one should be considered separately as well as the related action.

Report the session number and the related observation data in the same line. This attribution of session number validates the fact that data has been taken into count for compliance calculation.

Results per indication (indic) and per session (vertical):

4.1 Sum up indications per indication in the observation form: report the sum in the corresponding cell in the calculation form.

4.2 Sum up positive hand hygiene actions related to the total of indications above, making the difference between handwash (HW) and handrub (HR): report the sum in the corresponding cell in the calculation form.

4.3 Proceed in the same way for each session (observation form).

4.4 Add up all sums per each indication and put the calculation to calculate the ratio (given in percent)

***Note:** This calculation is not exactly a compliance result, as the denominator of the calculation is an indication instead of an opportunity. Action is artificially overestimated according to each indication. However, the result gives an overall idea of health-care worker’s behaviour towards each type of indication.

## Annexure 5. Chemoport insertion hands-on training log sheet.

**Name:_______________________________ Designation: _______________________**

**Table:**

Procedure: Chemoport Insertion – Observation			
**Sl.No**	**Name of Child**	**Supervised by**	**Signature**
			
			
			
			
			
			
			
			
			
			

**Table:**

Procedure: Chemoport Insertion – With supervision			
**Sl.No**	**Name of Child**	**HCP-2**	**Signature**
			
			
			
			
			
			
			
			
			
			

**Table:**

Procedure: Chemoport Insertion – Without supervision				
**Sl.No**	**Name of Child**	**Audited by**	**Score**	**Signature**
				
				
				
				
				
				
				
				
				
				

## Annexure 6. Process Indicators used to evaluate interventions planned.

**Table:**

Process indicators	Definition	Formula used	Tool used	Number of audits per month
PDSA 1:				
Adherence to the CCP	Adherence to CCP was said to be present if documentation in the rapid documentation sheet was complete.	Number of documentation sheets recorded completely/number of FN episodes X 100	-	All children with FN presenting to PER
Adherence to hand hygiene (HH)	HH was considered effective if the six essential steps of HH were followed for an appropriate duration.	No of moments where effective hand hygiene is performed/No of moments audited X 100	WHO hand hygiene audit tool (Annexure 4) https://cdn.who.int/media/docs/default-source/integrated-health-services-(ihs)/ssi/monitoring/observation-form.doc?sfvrsn=127f3133_2.	10 random moments of HH
Percentage of children receiving antibiotics through CVAD		Number of children receiving antibiotics through CVAD/Number of children with CVAD X 100	-	All episodes of FN among children with CVAD
Adherence to chemoport maintenance bundle	Adherence to 80% of the checklist was considered as safe CL maintenance	No of episodes of CL maintenance episodes with >80% adherence to CL maintenance bundle/No of episodes observed x 100	Department checklist*	At least 5 episodes of FN among children with CVAD
PDSA 2:				
Adherence to chemoport access bundle	Adherence to 80% of the checklist was considered as safe chemoport access	No of episodes of chemoport access with >80% adherence to bundle/No of episodes observed x 100	Department checklist*	Atleast 5 episodes of FN among children with chemoport

*Department checklist: A chemoport access & maintenance bundle checklist was developed and validated for the purpose of a QI study aimed at reducing CLABSI in the paediatric oncology unit (unpublished) was utilised

## References

[1] Ward ZJ, Yeh JM, Bhakta N (2019). Estimating the total incidence of global childhood cancer: a simulation-based analysis. Lancet Oncol.

[2] (2021). Clinicopathological Profile of Cancers in India: A Report of the Hospital-Based Cancer Registries.

[3] Ehrlich BS, McNeil MJ, Pham LTD (2023). Treatment-related mortality in children with cancer in low-income and middle-income countries: a systematic review and meta-analysis. Lancet Oncol.

[4] Agulnik A (2023). Management of septic shock in children with cancer - Common challenges and research priorities. J Pediatr (Rio J).

[5] Soeteman M, Potratz J, Nielsen JSA (2019). Research priorities in pediatric onco-critical care: an international Delphi consensus study. Intensive Care Med.

[6] Castagnola E, Fontana V, Caviglia I (2007). A prospective study on the epidemiology of febrile episodes during chemotherapy-induced neutropenia in children with cancer or after hemopoietic stem cell transplantation. Clin Infect Dis.

[7] Fletcher M, Hodgkiss H, Zhang S (2013). Prompt administration of antibiotics is associated with improved outcomes in febrile neutropenia in children with cancer. Pediatr Blood Cancer.

[8] Salstrom JL, Coughlin RL, Pool K (2015). Pediatric patients who receive antibiotics for fever and neutropenia in less than 60 min have decreased intensive care needs. Pediatr Blood Cancer.

[9] Lehrnbecher T, Robinson PD, Ammann RA (2023). Guideline for the management of fever and neutropenia in pediatric patients with cancer and hematopoietic cell transplantation recipients: 2023 update. J Clin Oncol.

[10] Koenig C, Schneider C, Morgan JE (2020). Association of time to antibiotics and clinical outcomes in patients with fever and neutropenia during chemotherapy for cancer: a systematic review. Support Care Cancer.

[11] Koenig C, Schneider C, Morgan JE (2020). Interventions aiming to reduce time to antibiotics (TTA) in patients with fever and neutropenia during chemotherapy for cancer (FN), a systematic review. Support Care Cancer.

[12] South African Triage Group (2008). A Division of the Emergency Medicine Society of South Africa.

[13] Freifeld AG, Bow EJ, Sepkowitz KA (2011). Clinical practice guideline for the use of antimicrobial agents in neutropenic patients with cancer: 2010 update by the Infectious Diseases Society of America. Clin Infect Dis.

[14] Raj R, Jain R, Kharya G (2022). Febrile Neutropenia. Standard Treatment Guidelines 2022.

[15] Gonzalez ML, Aristizabal P, Loera-Reyna A (2021). The golden hour: sustainability and clinical outcomes of adequate time to antibiotic administration in children with cancer and febrile neutropenia in Northwestern Mexico. JCO Global Oncol.

[16] Mccavit TL, Winick N (2012). Time-to-antibiotic administration as a quality of care measure in children with febrile neutropenia: a survey of pediatric oncology centers. Pediatr Blood Cancer.

[17] Kram DE, Salafian K, Reel SM (2020). A quality improvement initiative: Improving time-to-antibiotics for pediatric oncology patients with fever and suspected neutropenia. medRxiv.

[18] Lukes T, Schjodt K, Struwe L (2019). Implementation of a nursing based order set: improved antibiotic administration times for pediatric ED patients with therapy-induced neutropenia and fever. J Pediatr Nurs.

[19] Pole M, Blamires J, Dickinson A (2022). Improving the time to antibiotic administration in paediatric febrile neutropenia: implementation of a clinical care pathway in Saudi Arabia. Saudi J Nurs Health Care.

[20] Emerson BL, Prozora S, Jacob A (2019). An initiative to decrease time to antibiotics for patients with fever and neutropenia. Am J Med Qual.

[21] Al Sudairy R, Alzahrani M, Alkaiyat M (2019). Improving door-to-antibiotic administration time in patients with fever and suspected chemotherapy-induced neutropenia: a tertiary care center experience. Global J Qual Saf Healthcare.

[22] Todurkar N, Trehan A, Bansal D (2021). Time to antibiotic administration in children with febrile neutropenia: report from a low-middle-income country. Indian J Med Res.

[23] Zahan S, Ks R, Bhattachharyya P (2023). Door-to-needle time for administration of antibiotic in children with cancer within the golden hour- an audit from a tertiary cancer hospital in a low-middle income country. J Pediatric Infect Dis Soc.

[24] Mendieta A, Rios Lopez L, Vargas Arteaga M (2023). A multimodal strategy to improve health care for pediatric patients with cancer and fever in Peru. Rev Panam Salud Publica.

[25] Monroe K, Cohen CT, Whelan K (2018). Quality initiative to improve time to antibiotics for febrile pediatric patients with potential neutropenia. Pediatric Qual & Saf.

[26] Yoshida H, Leger KJ, Xu M (2018). Improving time to antibiotics for pediatric oncology patients with suspected infections: an emergency department-based quality improvement intervention. Pediatr Emerg Care.

[27] Spencer S, Nypaver M, Hebert K (2017). Successful emergency department interventions that reduce time to antibiotics in febrile pediatric cancer patients. BMJ Qual Improv Rep.

[28] Vanderway J, Vincent C, Walsh SM (2017). Implementation of a pathway for the treatment of fever and neutropenia in pediatric patients with cancer. J Pediatr Oncol Nurs.

[29] Benner CA, Mora E, Mueller E (2018). Making improvements in the ED: does ED busyness affect time to antibiotics in febrile pediatric oncology patients presenting to the emergency department?. Pediatr Emerg Care.

[30] Cohen C, King A, Lin CP (2016). Protocol for reducing time to antibiotics in pediatric patients presenting to an emergency department with fever and neutropenia: efficacy and barriers. Pediatr Emerg Care.

[31] Volpe D, Harrison S, Damian F (2012). Improving timeliness of antibiotic delivery for patients with fever and suspected neutropenia in a pediatric emergency department. Pediatrics.

[32] Koenig C, Kuehni CE, Bodmer N (2022). Time to antibiotics is unrelated to outcome in pediatric patients with fever in neutropenia presenting without severe disease during chemotherapy for cancer. Sci Rep.

[33] De Castro GC, Slatnick LR, Shannon M (2024). Impact of time-to-antibiotic delivery in pediatric patients with cancer presenting with febrile neutropenia. JCO Oncol Pract.

[34] Haeusler GM, Dashti SG, James F (2024). Impact of time to antibiotics on clinical outcome in paediatric febrile neutropenia: a target trial emulation of 1685 episodes. Lancet Reg Health Western Pac.

